# Synthesis and investigation of tetraphenyltetrabenzoporphyrins for electrocatalytic reduction of carbon dioxide

**DOI:** 10.1039/c8se00422f

**Published:** 2018-10-16

**Authors:** Dogukan H. Apaydin, Engelbert Portenkirchner, Pichayada Jintanalert, Matthias Strauss, Jirapong Luangchaiyaporn, Niyazi Serdar Sariciftci, Patchanita Thamyongkit

**Affiliations:** a Linz Institute for Organic Solar Cells (LIOS) , Institute of Physical Chemistry , Johannes Kepler University Linz , 4040 Linz , Austria . Email: dogukan.apaydin@jku.at ; Email: dogukanhazar.apaydin@ist.ac.at; b Institute of Physical Chemistry , University of Innsbruck , 6020 Innsbruck , Austria; c Department of Chemistry , Faculty of Science , Chulalongkorn University , 10330 Bangkok , Thailand; d Research Group on Materials for Clean Energy Production STAR , Department of Chemistry , Faculty of Science , Chulalongkorn University , 10330 Bangkok , Thailand

## Abstract

We report the synthesis and electrochemical properties of freebase tetraphenyltetrabenzoporphyrin and its complexes of Zn(ii), Co(ii), Ni(ii), Cu(ii) and Sn(iv) towards electrochemical reduction of carbon dioxide (CO_2_). Based on cyclic voltammetry, it is shown that central metals significantly affect the electrocatalytic performance in the reduction of CO_2_ in terms of reduction potential and catalytic current enhancement. At an applied potential of –1.90 V *vs.* an Ag/AgCl quasi reference electrode for 20 h, the electrocatalytic reduction of CO_2_ realized by Zn(ii)- and Cu(ii)-tetraphenyltetrabenzoporphyrins to carbon monoxide resulted in faradaic efficiencies of around 48% and 33%, respectively.

## Abstract 2

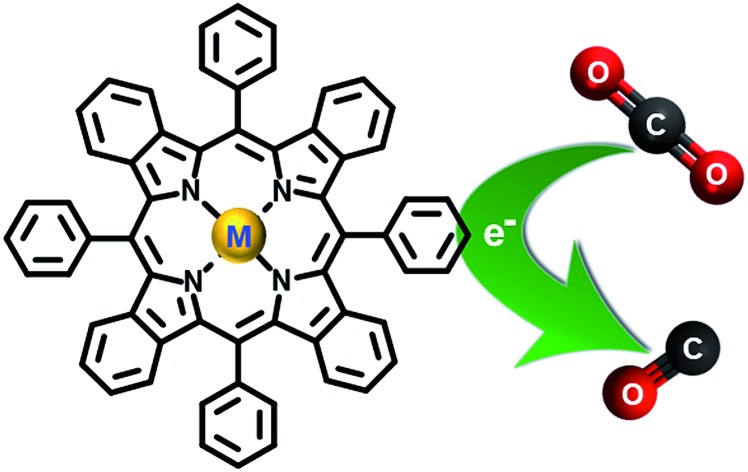
Benzoporphyrins with varying non-noble metal centers can reduce carbon dioxide to carbon monoxide with faradaic efficiencies changing between 33 and 48%.

## Introduction

Rapid global economic and population growth has led to an increase in fuel consumption in industrial, transportation, commercial and residential sectors. As a major source of energy, vast quantities of fossil fuels are burnt, creating environmental problems due to subsequent creation of greenhouse gases and pollutants. A greenhouse relevant product from fuel combustion processes is carbon dioxide (CO_2_). Therefore, assiduous scientific attention is directed towards identification of optimal methods to reduce the generation of CO_2_ and, at the same time, to convert CO_2_ into other useful compounds. One way to address this issue might be the cyclic use of carbon in human society, which stabilizes the CO_2_ content in the atmosphere.

The electrochemical reduction processes of CO_2_ using catalysts have attracted much attention because they require lower overpotential, compared to the direct reduction of CO_2_, and provide higher product selectivity.^1^–^5^ As economically friendly substitutes for precious-metal catalysts, organometallic electrocatalysts have become popular.^6^–^13^ Among such materials, porphyrin derivatives have been investigated continuously as potential electrochemical catalysts for the reduction of CO_2_.^14^,^15^ The great advantages of porphyrin compounds regarding this application originate from their high stability and tunability of their electrochemical properties by changing metal centers and substituents at the macrocyclic peripheral positions. Such structural modifications do not only enable improvement in the electrocatalytic performance of the systems, but also allow one to investigate the possible catalytic mechanisms.^16^–^19^

**Chart 1:**
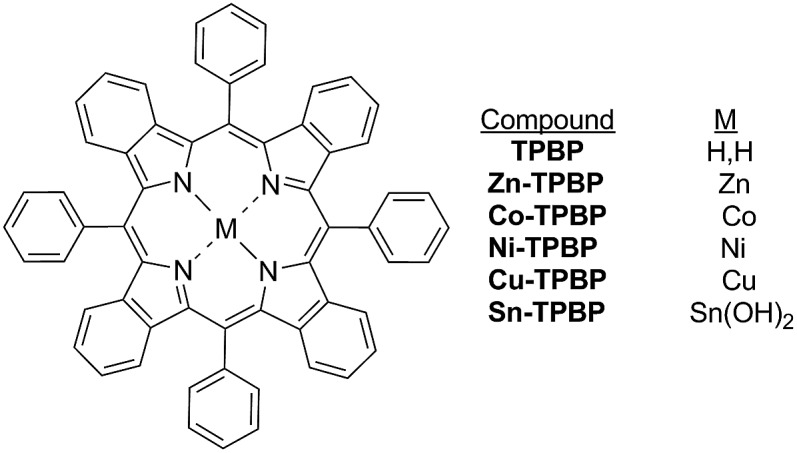
Chemical structure of the benzoporphyrins.

Tetrabenzoporphyrins are one of the very intriguing porphyrin derivatives that exhibit unique photophysical and electrochemical behaviors caused by extension of a π-conjugated structure at the β-positions of the porphyrin core and highly distorted macrocycle core due to *meso*-substitutions.^20^–^24^ Our previous work described systematic investigation of the photophysical and electrochemical properties of *meso*-substituted porphyrin and benzoporphyrin derivatives as ternary components for highly efficient bulk-heterojunction solar cells.^22^ Our observations on the electrochemical properties of the *meso*-substituted benzoporphyrin derivatives obtained from the previous study have led to extended investigation towards their catalytic activities for the electrochemical reduction of CO_2_ in this work. To the best of our knowledge, until now there are only a few studies on the electrochemical properties and catalysis of benzoporphyrin derivatives for CO_2_ reduction. Ramirez *et al.* described the electrochemical and photoelectrochemical reduction of CO_2_ using a Co(ii)-tetrabenzoporphyrin-modified electrode.^25^,^26^ To gain insights into the electrochemical properties of benzoporphyrins towards the electrochemical reduction of CO_2_, a series of metal-free and metallated tetraphenyltetrabenzoporphyrins as shown in Chart 1 were synthesized, characterized and investigated for their electrocatalytic activities for the reduction of CO_2_. Complexes of interest for this study had non-precious-metal centers like Zn(ii)–, Co(ii)–, Ni(ii)–, Cu(ii)– and Sn(iv)–, which were previously studied in porphyrin analogs for the electrochemical reduction of CO_2_.^27^–^31^ The overview presented in this work on the effect of the central metals on the electrochemical behavior and electrocatalytic activities of these benzoporphyrins for the reduction of CO_2_ will become a useful guideline for further development of several precious-metal-free oligopyrrole-based electrocatalytic systems.

## Results and discussion

### Synthesis

**Scheme 1:**
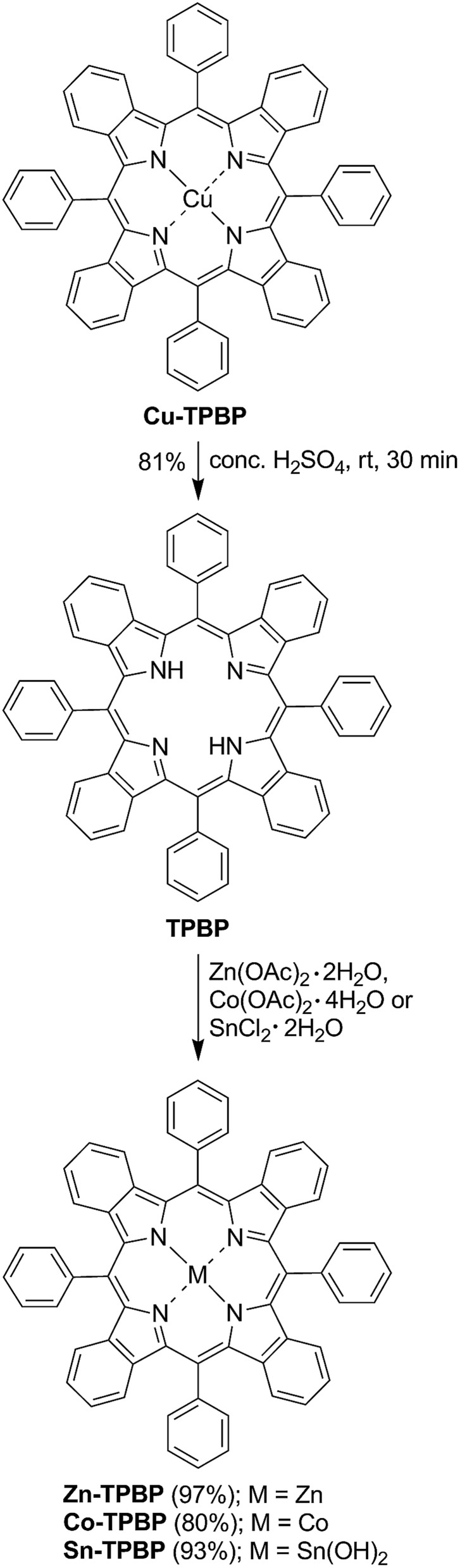
Synthesis of **Zn-TPBP**, **Co-TPBP** and **Sn-TPBP**.

Synthesis of the benzoporphyrins started from previously reported demetallation of **Cu-TPBP**^32^ by sulfuric acid at room temperature for 30 min, resulting in **TPBP** in 81% yield (Scheme 1).^33^ In its ^1^H-NMR spectrum, a broad singlet signal of two inner protons at *δ* –1.17 ppm confirmed the formation of **TPBP**. To obtain the desired metallated benzoporphyrins, **TPBP** was reacted with Zn(OAc)_2_·2H_2_O,^34^ Co(OAc)_2_·4H_2_O^35^ and SnCl_2_·2H_2_O^36^ as described in the previous studies, leading to **Zn-TPBP**, **Co-TPBP** and **Sn-TPBP**, respectively, in 80–97% yield. Complete metallation of **TPBP** in these reactions was monitored by the absence of the broad singlet signal of the benzoporphyrinic inner protons at *δ* –1.17 ppm in the ^1^H-NMR spectra and disappearance of the characteristic emission peak of **TPBP** at 787 nm. HR-ESI-MS confirmed the formation of both **Zn-TPBP** and **Co-TPBP** by showing their molecular ion peaks at *m*/*z* 876.2231 and 871.2273, respectively. Due to difficulties in chromatographic purification, **Sn-TPBP** could not be completely separated from other byproducts and was obtained in >90% purity based on ^1^H-NMR spectroscopy. However, its formation could be confirmed by MALDI-TOF MS showing its molecular ion peak at *m*/*z* 966.292. **Ni-TPBP** was obtained from a known procedure described in detail by Finikova *et al.*^32^

### Photophysical properties

**Fig. 1:**
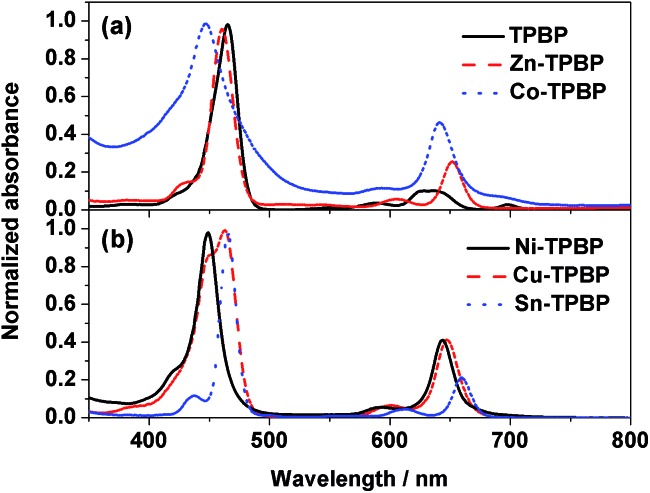
Normalized absorption spectra of (a) **TPBP** (black solid line), **Zn-TPBP** (red dashed line) and **Co-TPBP** (blue dotted line), and (b) **Ni-TPBP** (black solid line), and **Cu-TPBP** (red dashed line).

**Fig. 2:**
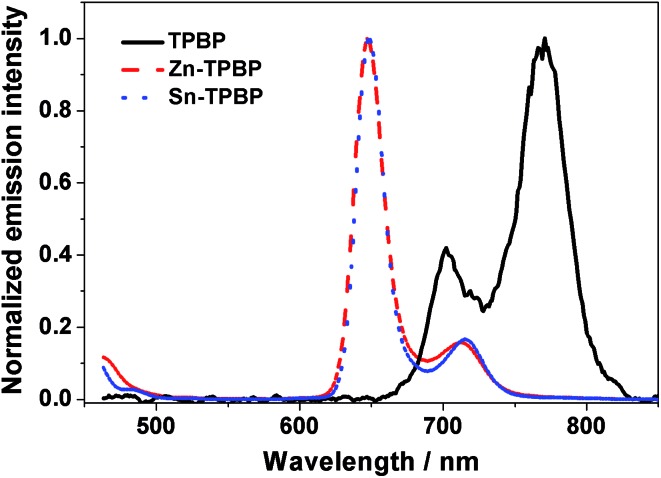
Normalized emission spectra of **TPBP** (black solid line), **Zn-TPBP** (red dashed line) and **Sn-TPBP** (blue dotted line).

As mentioned above, variation of the metal center of the benzoporhyrin derivatives does not only significantly affect the electrochemical behavior, but also the photophysical properties of the molecules. Therefore, absorption and emission of **TPBP**, **Zn-TPBP**, **Co-TPBP**, **Ni-TPBP**, **Cu-TPBP** and **Sn-TPBP** were investigated in this study to provide additional information that should be useful for further applications of these materials in optoelectronics and photoelectrocatalysis. By using UV-Vis spectrophotometry, normalized absorption spectra of all compounds in toluene were obtained as shown in Fig. 1. All compounds exhibited characteristic absorption patterns of the metallated benzoporphyrins having intense Soret bands in the range of 446–466 nm with absorption coefficients (*ε*) of 1.6 × 10^5^–5.3 × 10^5^ M^–1^ cm^–1^, and the Q-bands in the range of 592–698 nm, as summarized in Table 1. Upon excitation at their absorption maxima, **TPBP**, **Zn-TPBP** and **Sn-TPBP** showed emission peaks in the range of 658–787 nm as shown in Fig. 2 and Table 1, while **Ni-TPBP**, **Cu-TPBP** and **Co-TPBP** gave no significant emission.

**Table 1:** Absorption and emission spectral data of the benzoporphyrins

Compound	*λ*_abs_/nm (*ε* × 10^5^/M^–1^ cm^–1^)	*λ*_em_/nm
**TPBP**	465 (5.3), 588^*a*^, 626^*a*^, 640^*a*^, 698^*a*^	720, 787
**Zn-TPBP**	461 (2.8), 607^*a*^, 652^*a*^	658, 724
**Co-TPBP**	446 (1.7), 595^*a*^, 640 (0.8)	—^*b*^
**Ni-TPBP**	449 (2.2), 592^*a*^, 644 (0.9)	—^*b*^
**Cu-TPBP**	449 (1.6), 463 (1.9), 601 (0.1), 648 (0.8)	—^*b*^
**Sn-TPBP**	430 (0.3), 466 (4.1), 612^*a*^, 660 (0.9)	665, 745

^*a*^Due to low absorption, the *ε* value could not be determined.

^*b*^No emission peak was observed.

### Electrochemical characterization and catalytic activity

**Fig. 3:**
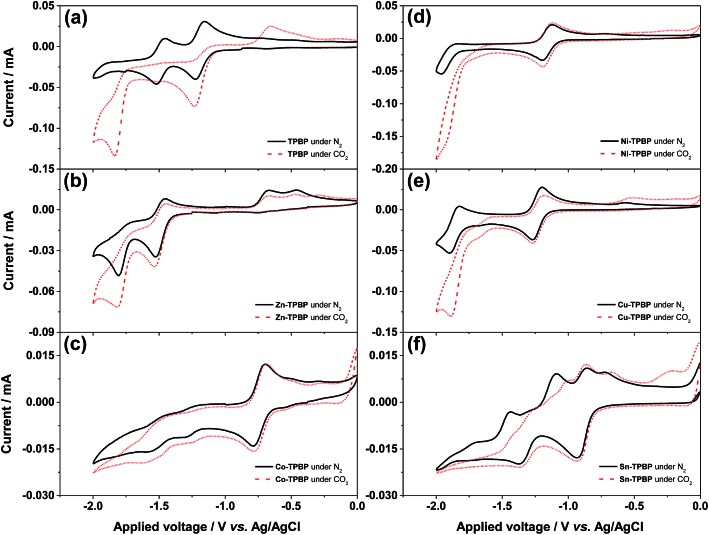
Cyclic voltammograms of a 0.1 M TBAPF_6_ solution in DMF containing 1.0 mM (a) **TPBP**, (b) **Zn-TPBP**, (c) **Co-TPBP**, (d) **Ni-TPBP**, (e) **Cu-TPBP** and (f) **Sn-TPBP** under the N_2_-(black solid line) and CO_2_-saturated (red dashed line) conditions recorded at a scan rate of 50 mV s^–1^ in the potential range of 0.00 to –2.00 V.

All benzoporphyrins were studied by means of cyclic voltammetry to evaluate their catalytic activities towards electrochemical CO_2_ reduction. The cyclic voltammograms were recorded in the potential range of 0.0 V to –2.0 V from a 0.1 M TBAPF_6_ solution in DMF containing 1.0 mM benzoporphyrin with a scan rate of 50 mV s^–1^ under the N_2_- and CO_2_-saturated conditions at ambient temperature and pressure. Fig. 3a shows that under the N_2_-saturated conditions, the first and the second reduction steps of **TPBP** are characterized by reversible reduction peaks at –1.22 V and –1.52 V, respectively. However, when the solution of **TPBP** was saturated with CO_2_, the first irreversible reduction was also observed at –1.22 V with a two-fold increase in peak current from 0.04 mA to 0.08 mA, while its second irreversible reduction peak appeared at –1.84 V with a negative shift by 0.32 V and a peak current enhancement from 0.05 mA to 0.13 mA. The great difference in the electrochemical behavior of **TPBP** observed in this case, compared with that under the N_2_-saturated conditions, may be attributed to possible binding between CO_2_ and inner pyrrolenine nitrogen atoms of the freebase benzoporphyrin macrocycle. The binding CO_2_ might form an ionic salt that is stabilized by the TBA cation which may present itself as a peak at more negative potentials. Moreover, new anodic signals were observed in the potential range of –0.40 V to –0.80 V, indicating the generation of unknown products from the irreversible reduction process(es) or the release of captured carbon dioxide. As for the electrochemical reduction of **Zn-TPBP** (Fig. 3b), a fully reversible first reduction (around –1.6 V) and as suggested by the scan-rate dependency of the peak current, a quasi-reversible second reduction peak (around –1.8 V) can be observed under N_2_-saturated conditions. A slight current increase for the first reduction peak and a two-fold current increase (from 0.05 mA to 0.10 mA) for the second one were monitored under CO_2_-saturated conditions signaling the reduction of CO_2_. Unknown oxidation signals were found under both N_2_- and CO_2_-saturated conditions in the potential range of –0.30 V to –0.70 V, which may be attributed to the side products formed during the reduction process. This observation of the increase in current and the change in the shape of the second ligand-based peak suggested that the doubly-reduced **Zn-TPBP** is the responsible molecule for the reduction of CO_2_.

The cyclic voltammogram of **Co-TPBP** in the N_2_-saturated solution revealed a reversible reduction peak at –0.75 V stemming from the metal-centered reduction of the molecule. This was followed by two quasi-reversible peaks at –1.29 V and –1.55 V which may be attributed to the multi-step ligand-centered reduction of the *meso*-substituted benzoporphyrin macrocycle (Fig. 3c). Under the CO_2_-saturated conditions, **Co-TPBP** showed a very similar reduction pattern with a slight current increase. Compared with planar porphyrin and phthalocyanine derivatives reported as potential catalysts.^37^ The difference in the catalytic behavior of **Co-TPBP** observed herein should stem from the unique saddle-shaped macrocycle distortion of the metallated benzoporphyrins previously studied by several groups.^20^–^24^

The cyclic voltammogram of **Ni-TPBP** under the N_2_-saturated conditions showed the first reversible reduction peak at –1.10 V which is ligand-centred and the second quasi-reversible one at –1.90 V that originates from metal-centered reduction.^38^ (Fig. 3d). Under the CO_2_-saturated conditions, similar electrochemical features were observed with a 3.6-fold current enhancement of the second reduction from 0.05 mA to 0.18 mA and a positive shift of its onset potential (*E*_onset_) by 0.20 V, suggesting the catalytic activity of **Ni-TPBP** towards the electrochemical reduction of CO_2_. In a similar manner, the N_2_-saturated solution of **Cu-TPBP** exhibited two major reversible reduction peaks at –1.27 V and –1.90 V which are attributed to the formation of a dianion radical and dianion of the macrocycle, respectively as shown in Fig. 3e.^38^,^39^ In the presence of CO_2_, a considerable current increase from 0.05 mA to 0.13 mA was observed at –1.85 V with a positive shift of *E*_onset_ by 0.18 V, suggesting the catalytic activity of **Cu-TPBP** for the electrochemical reduction of CO_2_. **Sn-TPBP** showed rather complex electrochemical behavior with two major reduction peaks at –0.92 V and –1.36 V, followed by at least five anodic peaks under the N_2_-saturated conditions, suggesting irreversible structural changes of the molecules upon the reduction processes (Fig. 3f). In the presence of CO_2_, its cyclic voltammogram also exhibited two main reduction peaks at a similar peak potential with even more complex anodic signals and a negligible current enhancement. This was attributed to possible interaction of the axial hydroxyl ligands of **Sn-TPBP** and CO_2_ that might lead to unexpected formation of side products upon reduction.

To further investigate the electrocatalytic performance of each benzoporphyrin towards the electrochemical reduction of CO_2_, all compounds were subjected to constant-potential electrolysis at an applied potential of –1.90 V for 20 h. The results from GC headspace analysis showed that the CO_2_-saturated electrolyte solutions containing **TPBP**, **Co-TPBP**, **Ni-TPBP** and **Sn-TPBP** did not yield any detectable reduction product. In the case of **TPBP**, it is likely that the above-mentioned binding between CO_2_ and the benzoporphyrin core was relatively strong and therefore suppressed the favorable reduction process of CO_2_ molecules. The low current enhancement observed for the above-described cyclic voltammograms of **Co-TPBP** and **Sn-TPBP** under the CO_2_ atmosphere could imply their low catalytic efficiencies for the electrochemical reduction of CO_2_. As for **Ni-TPBP**, although the current enhancement was found to be quite high in the cyclic voltammetry study, the reduction process did not lead to any detectable amount of CO or any other gaseous products. It is possible that the reduction process proceeded *via* other mechanisms and further optimization of the reduction conditions, such as addition of proton sources, pH adjustment and use of a co-catalyst, may be required. When **Zn-TPBP** and **Cu-TPBP** were used as catalysts, 8.67 μmol and 12 μmol of CO were detected, corresponding to a faradaic efficiency of 33% and 48% with turnover numbers of 1.7 and 6.6, respectively.

## Conclusions

Six tetraphenyltetrabenzoporphyrins, including the freebase derivative and the metal-chelated ones having Zn, Co, Ni, Cu and Sn metal centers, were successfully synthesized and characterized. According to UV-visible and fluorescence spectrophotometry, and cyclic voltammetry, the introduction and variation of the metal centers obviously affected the photophysical properties, electrochemical behaviors and catalytic activities for the electrochemical reduction of CO_2_ to CO. The results from cyclic voltammetry showed that, compared with the N_2_-saturated conditions, **TPBP**, **Ni-TPBP**, **Zn-TPBP** and **Cu-TPBP** exhibited a significant current enhancement, while **Co-TPBP** and **Sn-TPBP** gave only a slight increase in the peak current when CO_2_ was introduced into the solution. The constant-potential electrolysis revealed the formation of CO as the main reduction product with faradaic efficiencies of 33% and 48% and turnover numbers of 1.7 and 6.6 for **Zn-TPBP** and **Cu-TPBP**, respectively. This study suggests that, among the metallobenzoporphyrins of interest, Zn- and Cu-chelated analogs exhibited superior electrocatalytic activities towards the electrochemical reduction of CO_2_ to CO. Here, we show the catalytic potential of the metallated benzoporphyrins as alternative CO_2_ reduction catalysts using cheaper and more abundant metal centers like Zn and Cu, compared to Re and Pd. Optimization of the reduction conditions, together with structural modification of benzoporphyrin ligands will be further studied and described elsewhere.

## Experimental section

### Materials and methods

All chemicals were of analytical grade, purchased from commercial suppliers and used as received without further purification. ^1^H-NMR and ^13^C-NMR spectra were obtained in deuterated chloroform (CDCl_3_) at 400 megahertz (MHz) for ^1^H nuclei and 100 MHz for ^13^C nuclei. Chemical shifts (*δ*) are reported in parts per million (ppm) relative to the residual CHCl_3_ peak (7.26 ppm for ^1^H-NMR and 77.0 ppm for ^13^C-NMR spectroscopy). Mass spectra were obtained using high-resolution electron spray ionization mass spectrometry (HR-ESI-MS) and matrix-assisted laser desorption ionization-time of flight mass spectrometry (MALDI-TOF MS) with dithranol as a matrix. Ultraviolet-visible (UV-vis) and fluorescence spectrophotometry were performed in toluene at room temperature. Molar extinction coefficients (*ε*) are expressed in M^–1^ cm^–1^.

### Non-commercial compounds

*meso*-tetraphenyltetrabenzoporphyrinatonickel(ii) (**Ni-TPBP**)^32^ and *meso*-tetraphenyltetrabenzoporphyrinatocopper(ii) (**Cu-TPBP**)^32^ were prepared by a published procedure.

### Synthesis and characterization of benzoporphyrin derivatives

#### *meso*-Tetraphenyltetrabenzoporphyrin (**TPBP**)

Following a previously published procedure,^33^**Cu-TPBP**^32^ (0.052 g, 0.060 mmol) was reacted with concentrated sulfuric acid (10 mL) at room temperature for 30 min. The resulting reaction mixture was poured into a water/ice mixture and then extracted with dichloromethane. The combined organic phase was dried over anhydrous Na_2_SO_4_ and concentrated to dryness. The crude mixture was purified by column chromatography (silica gel, CH_2_Cl_2_/hexanes (2 : 1)) to afford **TPBP** as a green solid (0.039 g, 81%). ^1^H-NMR: *δ*_H_ –1.17 (s, 2H), 7.34–7.43 (m, 8H), 7.82–8.02 (m, 16H), 8.37 (d, *J* = 7.6 Hz, 4H), 8.56 (d, *J* = 7.2 Hz, 8H); ^13^C-NMR: *δ*_C_ 114.5, 115.8, 124.3, 124.7, 125.9, 128.6, 129.0, 129.3, 129.5, 130.1, 131.5, 134.7, 136.2, 139.9, 141.5, 142.1; MALDI-TOF-MS *m*/*z* (%): found 814.554 (100) [M^+^]; calcd 814.971 (M = C_60_H_38_N_4_); HR-ESI-MS *m*/*z*: [M + H]^+^ calcd for M = C_60_H_38_N_4_, 815.3175; found 815.3174; *λ*_abs_(*ε* × 10^5^) 465(5.3), 588, 626, 640, 698 nm; *λ*_em_ (*λ*_ex_ = 465 nm) 720, 787 nm.

#### *meso*-Tetraphenyltetrabenzoporphyrinatozinc (**Zn-TPBP**)

Following a previously published procedure,^34^ a solution of **TPBP** (0.131 g, 0.161 mmol) in chloroform (117 mL) was reacted with a solution of Zn(OAc)_2_·2H_2_O (0.177 g, 0.805 mmol) in methanol (13 mL) at room temperature for 12 h. After that, the resulting mixture was washed with water, and the organic layer was separated and dried over anhydrous Na_2_SO_4_. After removal of the solvent, the crude mixture was purified by column chromatography (silica gel, CH_2_Cl_2_/hexanes (2 : 1)) to afford **Zn-TPBP** as a greenish blue solid (0.136 g, 97%). ^1^H-NMR: *δ*_H_ 7.16 (dd, *J* = 6.0, 2.8 Hz, 8H), 7.28 (dd, *J* = 6.0, 2.8 Hz, 8H) 7.86 (t, *J* = 7.2 Hz, 8H), 7.93 (t, *J* = 7.2 Hz 4H), 8.30 (d, *J* = 7.2 Hz, 8H); ^13^C-NMR: *δ*_C_ 117.3, 124.3, 124.4, 125.5, 128.8, 129.0, 129.1, 132.7, 134.1, 134.2, 138.6, 143.2, 143.4; MALDI-TOF-MS *m*/*z* (%): found 875.913 (100) [M^+^], calcd 875.365 (M = C_60_H_36_N_4_Zn); HR-ESI-MS *m/z*: [M^+^] calcd for M = C_60_H_36_N_4_Zn, 876.2231; found 876.2231; *λ*_abs_(*ε* × 10^5^) 461(2.8), 607, 652 nm; *λ*_em_ (*λ*_ex_ = 461 nm) 658, 724 nm.

#### *meso*-Tetraphenyltetrabenzoporphyrinatocobalt(**Co-TPBP**)

Following a previously published procedure,^35^ a solution of **TPBP** (0.048 g, 0.059 mmol) in chloroform (45 mL) was reacted with a solution of Co(OAc)_2_·4H_2_O (0.073 g, 0.30 mmol) in methanol (5 mL) at room temperature for 4 h. After the mixture was washed with water, the organic phase was separated and dried over anhydrous Na_2_SO_4_. The solvent was removed and the resulting crude product was purified by column chromatography (silica gel, CH_2_Cl_2_/MeOH (99 : 1)) to afford **Co-TPBP** as a dark green solid (0.047 g, 89%). MALDI-TOF-MS *m*/*z* (%): found 870.594 (100) [M^+^], calcd 871.888 (M = C_60_H_36_N_4_Co); HR-ESI-MS *m*/*z*: [M^+^] calcd for M = C_60_H_36_N_4_Co, 871.2272; found 871.2273; *λ*_abs_(*ε* × 10^5^) 446(1.7), 595, 640(0.8) nm. Upon excitation at 446 nm, no emission peak was observed.

#### *meso*-Tetraphenyltetrabenzoporphyrinatotin (**Sn-TPBP**)

Following a previously published procedure,^36^ a solution of **TPBP** (0.102 g, 0.125 mmol) and SnCl_2_·2H_2_O (0.141 g, 0.625 mmol) in dimethylformamide (DMF, 5 mL) was treated with pyridine (0.05 mL) and refluxed for 4 h. A blue green precipitate was formed and collected by filtration. After that, the resulting crude product was purified by column chromatography (silica gel, CH_2_Cl_2_/MeOH (99 : 1)) to afford **Sn-TPBP** as a deep green solid (0.047 g, 90%). Due to incomplete purification of column chromatography, the achieved product was obtained with >90% purity, based on ^1^H-NMR spectroscopy. ^1^H-NMR: *δ*_H_ 7.23 (dd, *J* = 6.4, 3.2 Hz, 8H), 7.43 (dd, *J* = 6.4, 3.2 Hz, 8H) 7.90 (t, *J* = 7.6 Hz, 8H), 8.00 (t, *J* = 7.6 Hz 4H), 8.34 (d, *J* = 7.6 Hz, 8H); ^13^C-NMR: *δ*_C_ 116.5, 125.6, 127.2, 127.6, 129.7, 129.8, 133.9, 137.4, 137.6, 137.8, 141.3, 141.6, 141.7, 141.8, 142.0; MALDI-TOF-MS *m*/*z* (%): found 966.292 (100) [M^+^], calcd 965.679 (M = C_60_H_38_N_4_O_2_Sn); *λ*_abs_(*ε* × 10^5^) 430(0.3), 466(4.1), 612, 660(0.9) nm; *λ*_em_ (*λ*_ex_ = 466 nm) 665, 745 nm.

### Electrochemical studies

#### Background current

A background cyclic voltammogram was obtained in an anhydrous solution (10 mL) of 0.1 M tetrabutylammonium hexafluorophosphate (TBAPF_6_) in DMF. A three-electrode one-compartment cell was used throughout the experiments. A glassy carbon electrode served as a working electrode while a Pt plate was used as a counter electrode. A silver wire coated with silver chloride (Ag/AgCl) was used as a quasi-reference electrode (QRE). The Ag/AgCl QRE was prepared using a procedure described elsewhere^40^ and externally calibrated with a ferrocene/ferrocenium redox couple using a potential of 0.72 V *vs.* a normal hydrogen electrode (NHE) as a reference value.^41^ The cyclic voltammograms were recorded at potentials ranging from 0.00 V to –2.00 V *vs.* a Ag/AgCl QRE at a scan rate of 50 mV s^–1^. The potential values were therefore reported with respect to the Ag/AgCl QRE. The solution was purged with nitrogen (N_2_) or CO_2_ for 20 min before each measurement with a flow rate of 0.2 L min^–1^. As a control experiment, constant-potential electrolysis of the electrolyte solution under CO_2_-saturated conditions in the absence of the catalyst for 20 h did not yield any CO_2_ reduction product.

### Electrochemical reduction of CO_2_

The catalytic activity of the benzoporphyrins towards electrochemical reduction of CO_2_ was investigated by means of cyclic voltammetry and the constant-potential electrolysis using the above-mentioned electrochemical setup at potentials ranging from 0.00 to –2.00 V. Each cyclic voltammogram was collected from a 0.1 M TBAPF_6_ solution in anhydrous DMF containing 1.0 mM benzoporphyrin (10 mL) at a scan rate of 50 mV s^–1^ under N_2_- or CO_2_-saturated conditions. The solution was purged with N_2_ or CO_2_ for 20 min before each measurement with a flow rate of 0.2 L min^–1^. The constant-potential electrolysis of each benzoporphyrin was performed at ambient temperature using the above-mentioned electrochemical setup and at an applied potential of –1.90 V. After 20 h, a 2 mL gas sample from headspace gas (total volume was 10 mL) was taken from a reaction vial and analyzed by gas chromatography (GC) equipped with a thermal conductivity detector (TCD). Curves for CO obtained from the experiments were integrated to get the peak area, which was then used to calculate the amount of CO in the headspace using a pre-measured calibration curve.

## Conflicts of interest

There are not conflicts to declare.
